# Guiding Chart for Initial Layer Choice with Nanoimprint Lithography

**DOI:** 10.3390/nano11030710

**Published:** 2021-03-11

**Authors:** Andre Mayer, Hella-Christin Scheer

**Affiliations:** 1 Chair for Large Area Optoelectronics, School of Electrical, Information and Media Engineering, University of Wuppertal, Rainer-Gruenter-Str. 21, 42119 Wuppertal, Germany; 2 School of Electrical, Information and Media Engineering, University of Wuppertal, Rainer-Gruenter-Str. 21, 42119 Wuppertal, Germany; scheer@uni-wuppertal.de

**Keywords:** nanoimprint lithography, negligible residual layer, partial cavity filling, guiding chart, defect avoidance, hydrodynamic instabilities, T-NIL, UV-NIL, el-UV-NIL, el-T-NIL

## Abstract

When nanoimprint serves as a lithography process, it is most attractive for the ability to overcome the typical residual layer remaining without the need for etching. Then, ‘partial cavity filling’ is an efficient strategy to provide a negligible residual layer. However, this strategy requires an adequate choice of the initial layer thickness to work without defects. To promote the application of this strategy we provide a ‘guiding chart’ for initial layer choice. Due to volume conservation of the imprint polymer this guiding chart has to consider the geometric parameters of the stamp, where the polymer fills the cavities only up to a certain height, building a meniscus at its top. Furthermore, defects that may develop during the imprint due to some instability of the polymer within the cavity have to be avoided; with nanoimprint, the main instabilities are caused by van der Waals forces, temperature gradients, and electrostatic fields. Moreover, practical aspects such as a minimum polymer height required for a subsequent etching of the substrate come into play. With periodic stamp structures the guiding chart provided will indicate a window for defect-free processing considering all these limitations. As some of the relevant factors are system-specific, the user has to construct his own guiding chart in praxis, tailor-made to his particular imprint situation. To facilitate this task, all theoretical results required are presented in a graphical form, so that the quantities required can simply be read from these graphs. By means of examples, the implications of the guiding chart with respect to the choice of the initial layer are discussed with typical imprint scenarios, nanoimprint at room temperature, at elevated temperature, and under electrostatic forces. With periodic structures, the guiding chart represents a powerful and straightforward tool to avoid defects in praxis, without in-depth knowledge of the underlying physics.

## 1. Introduction

The basic idea behind nanoimprint was to propose a low-cost alternative for sub-micrometer lithography [1,2,3] without the need for highly sophisticated vacuum equipment. This intended application is expressed in its abbreviation, NIL (nanoimprint lithography). Typically, with lithography applications a thin polymeric layer on a hard substrate is imprinted. Meanwhile NIL has entered a much broader field of applications and is most often used for patterning the surface of polymeric substrates and foils. Surface structures may provide a specific wetting/de-wetting behavior for liquid phases [4]; surface structures also have a wide range of applications in optics, e.g., as gratings and anti-reflective, wave-guiding, or feedback structures [5,6,7]. In these cases, nanoimprint often does not involve thin layers on a hard substrate, and is therefore similar to hot embossing [8], a technique matured in the field of MEMS (micro-electro mechanical systems). Nanoimprint was reviewed with respect to a number of different aspects [9,10,11,12,13,14,15,16,17,18,19].

**Figure 1:**
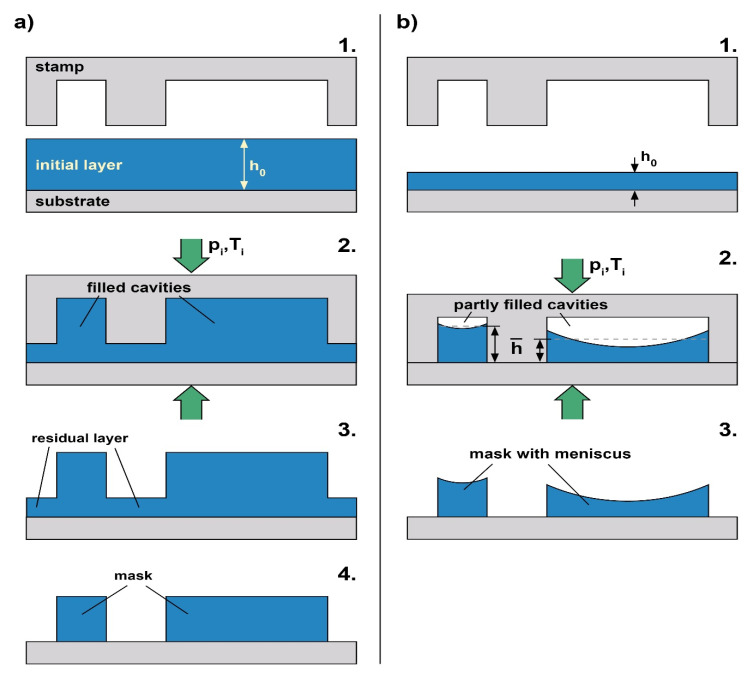
Imprint strategies with nanoimprint lithography (NIL) as a lithography process to provide a mask for the subsequent patterning of a substrate (*p_i_* = imprint pressure, *T_i_* = imprint temperature). (**a**) Conventional imprint with a residual layer remaining and filled cavities. (**b**) Concept of ‘partial cavity filling’ (idealized); all cavities are under-filled, and the polymer adopts a horizontal meniscus between the stamp sidewalls. 1. Initial situation with polymer height *h*_0_. 2. Imprinted situation with/without residual layer and fully/partly filled cavities. 3. Polymeric structures after stamp removal, connected/isolated. 4. Polymeric structures after residual layer removal, with conventional imprint (**a**).

Here, we address nanoimprint as a lithography technique, employed to provide a local mask with isolated structures that define windows for further processing to the substrate underneath. With optical lithography, these windows result in a straightforward way by complete removal of the photoresist in exposed regions during development. However, with NIL, this is not naturally the case due to the residual layer, typically remaining with T-NIL (thermal NIL [1,10,20,21,22,23]) and with UV-NIL (ultraviolet-assisted NIL [2,3,24,25,26,27]) as well. Without specific precautions, a certain amount of imprint material always remains below the elevated stamp structures (see Figure 1a). With NIL it is common practice to remove this residual layer, e.g., by dry etching to obtain structures isolated from each other (Figure 1(a4)). An effective removal of the residual layer by dry etching asks for a high uniformity of the imprint to avoid a lateral loss of the masking structures during this step.

Some attempts were reported to imprint directly without a residual layer remaining to avoid such an intermediate etching step. With radiation-sensitive materials (curable materials, negative tone photoresists) just this property offers a way of lending itself to avoid etching. With UV-NIL, when the elevated structures of the stamp are provided with e.g., a metal, the residual layer is not cured and can simply be removed in a suitable solvent [28,29,30]. With T-NIL of photoresists, during flood exposure following imprint standing wave effects may simply be used to avoid curing of a thin residual layer [31]. In both cases the main limitation is a geometric one; the residual layer has to be thin and the stamp structures should be wider than the diffraction limit of the exposing radiation. 

However, to achieve a thin residual layer is not trivial, in particular not over large areas. Due to the mechanical nature of nanoimprint, with UV-NIL and T-NIL as well, a uniform residual layer can only be obtained with periodic structures all over the stamp. With a non-periodic stamp either the structure height or the residual layer is non-uniform by itself [32]. The decisive factor in this context is the thickness of the stamp used. When the stamp is thick it is rigid enough to prohibit bending; then the residual layer is uniform but the structure height varies as some of the stamp cavities remain only partly filled. In contrast, with stamps that are thin or rather flexible, stamp bending may enable a complete filling of the cavities, however, then the residual layer becomes non-uniform [27,33,34,35,36]. With both non-uniformities, a varying structure height or a varying layer thickness, residual layer removal without structure loss is demanding [37].

Basically, any non-uniformity with locally varying lateral stamp geometries (the sizes of the cavities and the adjacent elevated stamp structures) results from a local filling of cavities [38]; any locally filled cavity impedes a further imprint in its neighborhood as the polymer retains its volume (compression negligible). In direct consequence, either the filling height (and thus the structure height) or the residual layer thickness (or both) become non-uniform. Hence, it is straightforward to avoid local cavity filling. One approach makes use of a stamp that locally provides additional cavity volume to equalize the residual layer, the so-called ‘capacity equalized mold’ [39]. In this approach, the stamp is prepared with differing cavity depths. Then, similar to the case of a rigid stamp, the residual layer is uniform, but the structure height varies. A simpler (and most efficient) approach with a constant cavity depth pursued in some groups is ‘partial cavity filling’, reported with soft stamps [40,41] and rigid stamps as well [29,42]. As sketched in Figure 1b, with ‘partial cavity filling’, the initial layer of the polymer is chosen so thin that the cavities remain under-filled with all stamp geometries involved. Again, the filling heights of the cavities and thus the structure heights then differ, but any residual layer can be largely avoided as (except for a few nanometers remaining [43]) the layer is imprinted through—basically the elevated stamp structures are in contact with the substrate at the end of the imprint (Figure 1(b2)). Stamp bending is avoided as well, as no cavity filling occurs. The method works well with stamps featuring a duty cycle (elevated stamp regions per area compared to cavities per area) that does not vary too much across the whole stamp, as it is the case with largely periodic structures. 

Unfortunately, the partial filling of cavities implies an additional risk, namely the formation of defects via thermodynamic instabilities [40,44]. They are observed with viscosities low enough to allow their development within the actual processing time. With instabilities, the polymer within the cavities develops sub-structures with periodicities depending on the local filling height of the cavities [45,46]. The resulting defects are well-known to the experimentalist with T-NIL and UV-NIL as well [20,21,47,48,49]. It was proposed to make use of such defects for patterning of the polymer [48,50,51,52,53,54], however, as the control of the lateral dimensions and the periodicity over large areas is limited, such attempts are restricted to the lab scale. In order to apply nanoimprint as a lithography technique, defects caused by instabilities ultimately have to be avoided, they represent mask defects.

The present investigation aiming at residual layer-free imprint applies the strategy of ‘partial cavity filling’, where the polymeric structures feature a horizontal meniscus as sketched in Figure 1(b2,3). The key issue in this context is an adequate choice of the initial layer thickness to provide isolated structures. However, in order to achieve a continuous meniscus this choice has to consider the stamp geometries (stamp height/cavity depth, duty cycle) and their interplay with the contact angle that the polymer develops with respect to the stamp; this first issue is a mainly geometric one. Furthermore, to avoid defects developing with time due to instabilities, the viscosity of the imprint material at processing conditions (with UV-NIL at room temperature, with T-NIL at elevated temperature) also has to be included in the considerations; this second issue is a thermodynamic one. It is our intention to mitigate these complex geometric/physical relationships and to render them manageable for practical use. Therefore, we provide a tool for such an adequate choice of the initial layer thickness, a ‘guiding chart’. This guiding chart indicates a window for defect-free processing by means of specific boundaries derived from the underlying physics. In our case, the boundaries are given in terms of the mean filling height and the cavity width, for a fixed height and duty cycle of the stamp. In contrast to attempts referring to soft molds [55,56], our approach is independent from the stamp material used and, in particular, considers thermally and electrically induced instabilities resulting from interaction of the imprint polymer with the ceiling of the stamp cavities.

To pave the way for a defect-free imprint with negligible residual layer thickness in praxis, it is essential to alleviate the complex physics of instabilities. To this end we will proceed as follows. (i) Where possible, we will work with generalized material properties and (ii) we will represent all theoretical relationships by diagrams from which the user is able to construct his own individual guiding chart (matching his specific imprint situation) in a straightforward way, without the need for a deep insight into physical relationships. We explain the procedure for constructing the guiding chart and discuss the processing window applying with T-NIL and UV-NIL, with and without electrostatic forces; by means of examples for these imprint techniques we propose an adequate choice of the initial layer thickness to avoid defects. Our simplifying approach is inspired by the characteristics of typical instability-induced defects observed with T-NIL. Starting with a guiding chart for periodic stamps with linear cavities, we will then broaden its applicability to cover periodic stamps with dot-like structures.

Our guiding chart provides an easily manageable tool to make use of otherwise rather complex physical relationships in praxis.

## 2. Materials and Methods

Typically, under processing conditions the materials to be processed via NIL are referred to as viscous liquids. Thus, their primary property is the viscosity *(η)*, determining the time-scale of material response. Moreover, the surface tension/surface energy *(γ)* of the imprint material and the stamp as well are of interest as they define the contact angle that develops between the viscous liquid and its boundaries inside the partly filled cavities under equilibrium conditions, after a sufficiently long interaction time (e.g., 90 min with our material at 190 °C, to be on the safe side). The surface energy of the substrate is of minor importance here.

Experimental evidence is provided by T-NIL with PS (polystyrene) as the imprint material. Beyond the data for our specific polymer, we indicate general trends and correlations to enable assessing other imprint materials. Moreover, typical data with materials suitable for UV-NIL are included as the application of our guiding chart is independent from the specific NIL technique used.

As stated, the T-NIL experiments were performed with PS as the imprint material. The PS used here (350 kg/mol, Sigma Aldrich, Darmstadt, Germany) is well characterized with respect to imprint-related properties. Its glass transition temperature is 95 °C [57]. According to GPC-analysis (gel permeation chromatography), its molar mass values amount to *M_n_* = 130 kg/mol (number average), *M_w_* = 280 kg/mol (weight average), *M_w_/M_n_* = 2.1 (poly-dispersity) [58]; polymers with such a wide molar mass distribution are relatively easy to imprint, however their zero shear to shear-thinning transition is less pronounced than with polymers featuring a low poly-dispersity. Dynamic mechanical analysis was carried out in a plate-plate geometry to determine viscosity master curves; Table 1 summarizes the viscosities of our PS at specific processing temperatures. Please note that the correlation between temperature and viscosity depends on the molar mass of the polymer (*η* increases with increasing molar mass) [58,59]; in addition, it depends on the chemical structure of the monomer; PMMA (polymethylmethacrylate) of a similar molar mass has a higher viscosity than PS [60]. With a polymeric material given, temperature acts as the control parameter to tune the viscosity during processing, the material parameter of interest in praxis. Viscosities near 10^4^ Pas and below are prone for instability-induced defects within 5 min of imprint time [57].

As further stated, the contact angle *θ* of the polymer within a partly filled cavity results from the surface energy/tension. The surface tension *γ_p_* of the polymer decreases with increasing temperature [61]. Typical imprint materials have surface tensions of 30–40 mJ/m^2^ at room temperature, decreasing by up to 10 mJ/m^2^ at typical imprint temperatures with T-NIL. Surface tensions characteristic with our PS are also given in Table 1, together with the contact angle *θ* the polymer develops to the stamp walls. Crucial in this respect is the quality of the anti-sticking layer of the stamp. We assumed a surface energy of 10 mJ/m^2^ and 15 mJ/m^2^ (being largely independent from temperature) as typical with an anti-sticking layer of excellent or good quality that is required for a defect-free separation of the stamp from the imprinted sample. The third value considered, ≈20 mJ/m^2^, refers to an anti-sticking layer of limited quality; this value indeed applies to a flexible stamp with a patterned surface layer from the elastomer PDMS (polydimethylsiloxane) as sometimes used with UV-NIL [17,19,40,62].

Table 1 indicates to what extent our characteristic material parameters vary with temperature. Typical viscosities effective with T-NIL are in the range 10^3^ Pas ≤ *η_p_* ≤ 10^5^ Pas, with all polymers; imprint materials differing from our specific PS (e.g., PMMA) may show these viscosities at a different temperature. Typical contact angles with T-NIL are in the range of 70° ≤ *θ* ≤ 80°.

For comparison, with UV-NIL viscosities are orders of magnitude lower during imprint at low pressure, in the range of 10^−1^–10^2^ Pas (e.g., epoxy silicone monomers [24] or other typical UV-NIL materials [26]); due to room temperature processing the surface tension and the contact angles are somewhat higher than with T-NIL (*γ_p_* ≈ 30–40 mJ/m^2^, *θ* ≈ 81–91°/66–77°/51–66°).

For the T-NIL experiments, layers of different thickness were spin-coated to Si substrates from a toluene solution and dried (120 °C, 10 min, hotplate). Imprint was performed with Si stamps (2 cm × 2 cm) provided with an anti-sticking layer [63]. The stamps are almost completely patterned; they contain test structures, fields of lines, and spaces with different geometries. The parallel-plate imprint system used [64,65] is equipped with electrical heating; heating to the imprint temperature takes about 15–25 min; after about 5 min the glass transition temperature is reached. Cool-down to below glass temperature occurs within ≤2 min due to efficient water cooling. The imprint pressure is 100 bar, and a typical imprint time is 5 min. Inspection of the samples after sputtering with Au was done by scanning electron microscopy (SEM, S-FEG XL 30 S, Thermo Fisher Scientific, Waltham, MA, USA).

## 3. Typical Experimental Results

**Figure 2:**
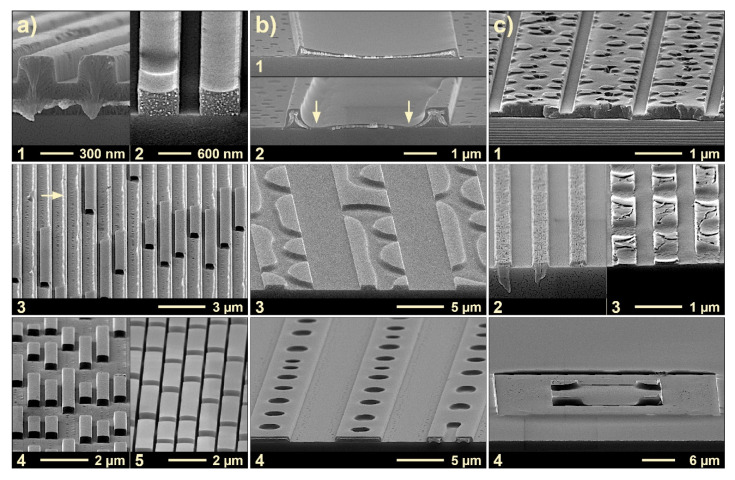
Examples obtained with T-NIL at differing initial layer thickness *h*_0_. If not stated otherwise in the text, the imprinted material is PS, and the imprint temperature is 190 °C. (**a**) Examples with narrow cavities (*w* = 300–800 nm). (**b**) Examples with wider cavities (*w* ≈ 5 µm). (**c**) Miscellaneous examples see text. The initial layer thickness increases from (**a3**) to (**a5**) and from (**b2**) to (**b4**).

Figure 2 gives examples of typical imprint results obtained with T-NIL when the initial layer thickness *h*_0_ is varied. Ideally, the imprinted polymeric structures should mirror the lateral stamp geometries, with a continuous horizontal meniscus at their top (see Figure 1(b2,3)). In praxis, when the initial layer thickness is not adequate, defects are observed, with polymeric structures smaller than the lateral dimensions of the cavities. In addition to defect-free structures, Figure 2 gives examples of defects characteristic of different cavity sizes. When the polymer is accumulated near an edge or corner the defect is geometry-related; when the polymeric sub-structures feature some periodicity, they result from instabilities. With narrow cavities, instabilities develop mainly along the length of the cavities; with wider geometries they may develop across the cavities as well. The examples mainly serve to illustrate nanoimprint under ‘partial cavity filling’ conditions to provide residual layer-free structures for lithography.

Most of the examples were obtained at an imprint temperature of 190 °C, where the viscosity of the PS used is low enough to allow the polymer that is squeezed into the cavity from the sides to adopt an equilibrium state within the partly filled cavities. This equilibrium state is characterized by a typical contact angle of the polymer towards the stamp (70°–80°). Though the examples were obtained with T-NIL only, they are similarly typical of results to be obtained with UV-NIL at room temperature, merely at much lower viscosities (and low driving forces/pressures) [2,3,24,25,26,40,66].

Figure 2(a1) shows an example with complete filling of the cavities, all other examples refer to ‘partial cavity filling’ conditions. Column (a) gives examples with narrow cavities, *w* ≈ 300–800 nm; column (b) gives examples with wider cavities, *w* ≈ 5 µm, and column (c) refers to differing specific situations. Most of the examples verify the existence of instabilities of the polymer within the cavity as some periodicity can be identified.

When the stamp cavities are completely filled, a residual layer typically remains below the imprinted structures, as clearly visible with (a1). When the residual layer is high compared to the structure height and when it is non-uniform (as it would be the case with locally differing duty cycle), residual layer removal by dry etching is challenging. In contrast, structures without a residual layer could serve as a mask directly, without an intermediate etching step.

With ‘partial cavity filling’ the most regular shape is the one of a continuous horizontal meniscus between the stamp sidewalls as documented in Figure 2(a2,b1), taking the example of a narrow and a wide cavity. When the masking height (the center height of the meniscus in the cavity) is sufficient, the structures with a horizontal meniscus are those that are well-suited to serve as a mask for etching of the substrate (e.g., a2); this is why structures with a continuous horizontal meniscus (see Figure 1(b2,3)) are the target structures for lithography applications of NIL (the masking height required depends on the mask selectivity of the etching process applied). With a similar mean filling level a sufficient masking height in the cavity center is easier to obtain with a smaller cavity, due to the spherical shape of the meniscus (see e.g., Figure 2(a2) compared to Figure 2(b1)).

Figure 2(b2–4) gives examples, where the meniscus formed involves the stamp ceiling rather than the stamp sidewalls. This occurs when, during meniscus formation, the contact line of the polymer at the sidewalls reaches the stamp corner. Then a rim forms all along the sidewalls, where the polymer fills the stamp cavity locally to its full height (b2); the rim increases in width when the initial layer thickness increases. As the formation of the rim results in a withdrawal of polymer from the neighborhood (see arrows) the masking height there may become small locally and result in de-wetting, precluding the use of such structures for lithography in praxis. Though (b2) results from purely geometric reasons, the fluctuations of the rim along the length of the cavity already indicate the onset of instabilities. Figure 2(b3,4)) illustrates the situation when the initial layer height is increased (compared to b2), thus increasing the mean filling level in the cavity. The periodicity of alternating filled and empty regions observed now is typical of fully developed instabilities, applying to the width and the length of the cavity. Again, these structures are unsuitable for lithography as they do not mirror the lateral stamp geometries. At relatively high filling level (b4) often a series of holes remains along the center of the cavity. If these holes were surface-near only so that a sufficient height of polymer remained below them for subsequent etching, such structures could serve as a mask. However, as the contact angle is below 90° with nanoimprint, these holes widen to the bottom; in addition, some randomness exists. Therefore, also polymeric structures similar to (b4) are too risky for lithography applications.

With narrow cavities (a3–5), the transition from a purely geometric effect, the polymer-stamp contact at the edge of the stamp cavity, to fully developed instabilities are less obvious. The cavities are either completely filled or empty, alternating along the length of the cavity, only. With a high mean filling level, the ‘on-off’-ratio is high (a4,5); (a3) is a rare example where the rim along the sidewalls of the stamp is still visible (see arrow), between regions of complete stamp filling (the stamp was not parallel to the substrate). Of course, these structures are far from being qualified for lithography purposes, where masking of the substrate along the complete length of the lines would be asked.

Figure 2(c1) refers to a specific situation where the imprint still shows a residual layer but the cavities are not completely filled (the stamp was held at a certain distance due to pattern size effects [67]. Within the small gap between the polymer surface and the stamp ceiling, periodic instabilities are visible. These small differences in polymer height are not restrictive for lithography, even when some residual layer has to be removed first, as it is the case here.

Figure 2(c2,3) refers to a thermal imprint under electrostatic forces. Here a lamellar block-copolymer was imprinted (PS-PMMA) under ‘partial cavity filling’ conditions, where a voltage was applied to induce a vertical phase separation in the block-copolymer. The situation in (c2) refers to a low imprint temperature (170 °C); instabilities did not occur. In contrast, instabilities along the cavities are fully developed when imprinting at high temperature, 210 °C (c3). In both cases the PMMA-part of the block-copolymer was removed, so that the PS-component remained, only.

Finally, Figure 2(c4) refers to a wide cavity of square shape (with a thick wall), located in the center of the micrograph. The polymer has filled the stamp cavity at the inner edges of the cavity only; any periodicity from instabilities is not visible. This results from a mainly geometric effect. Similar to the situation with a linear cavity (b2), a 2-dimensional cavity features the highest polymer level at the inner edges (due to meniscus formation in two directions). There, the polymer filled the cavity in full height, under withdrawal of material from the rest of the cavity. Again, suitability for lithography is precluded as the rest of the cavity is not masked.

From these examples observed in praxis we draw the following conclusions. With ‘partial cavity filling’, the meniscus at the polymer surface plays a central role for formation of intact or defective structures. The meniscus is controlled by the contact angle *θ* the polymer develops to the stamp. As long as a continuous horizontal meniscus forms between the stamp sidewalls, the polymeric structures obtained are well-suited for lithography purposes when their minimum height is sufficient for subsequent etching. This is the case when the polymer develops its contact angle to the sidewalls of the stamp, at mean filling levels of adequate height.

However, at a filling level too high the meniscus touches the ceiling of the stamp at the edges of the cavity. This results in a sort of ‘mode jump’, from a horizontal meniscus to a vertical meniscus. Now the polymer tends to develop its contact angle to the horizontal ceiling of the stamp cavity (and to the substrate). As a consequence, a reconfiguration of the polymeric liquid within the cavity occurs. This may lead to local defects where the substrate becomes exposed (de-wetting, see arrows in b2), rendering these polymer structures inapplicable to lithography, similar to the structures in b4. 

As will be addressed in part 4, instabilities may induce this mode jump at even lower filling levels. With instabilities, the polymer layer breaks down into more or less periodic sub-structures, characterized by a local filling of the stamp cavity to its full height alternating with a vanishing height. Depending on the lateral dimensions of the cavity and the characteristic periodicity, the reconfiguration of the polymer within the cavity may affect both geometries, its width, and its length. Though fascinating from a physical perspective instability-induced structures are less useful in praxis as some randomness over large areas cannot be avoided. In particular they are fully inappropriate for lithography purposes with nanoimprint as they do not mirror the lateral stamp geometries. 

Generally, the onset of instabilities at inadequate filling levels limits the use of ‘partial cavity filling’ for nanoimprint with negligible residual layer. This holds with a mean filling level too high (as exemplified in Figure 2) and too low as well; the latter may lead to de-wetting in the center of wide cavities.

As a consequence, only structures with a continuous horizontal meniscus are able to mask the substrate. The process window to be defined thus refers to the regime of the initial polymer height *h*_0_ where such a horizontal meniscus forms when a stamp of height H and with certain geometries of the elevated structures (*s*) and the cavities (*w*) is imprinted into the thin layer. The intention of nanoimprint with ‘partial cavity filling’ is to provide such structures without a residual layer, isolated from each other. Its realization requires an adequate choice of the initial layer thickness.

In the following, we will address the limitations imposed on ‘partial cavity filling’. We will start with purely geometric limitations (similar to b2 and c4) and present a preliminary guiding chart to identify a basic processing window for defect-free imprint (part 4), indicating a maximum cavity width allowed. Beyond imprint-specific aspects, application-specific aspects will also be addressed. This geometric guiding chart will then be modified to include instabilities (part 5). Finally, the construction of a specific guiding chart for practical application is addressed (part 6). Consequences for the choice of the initial layer are drawn and potential measures to widen the processing window are indicated.

## 4. Geometric Processing Window

A basic, purely geometric processing window can be identified from a guiding chart in a plane that is spanned by the mean filling height in the cavity, $\bar{h}$, and the cavity width, *w*. Further geometry parameters and material parameters involved are the stamp height *H*, the size of the elevated stamp structures *s* (or rather their duty cycle *s/w*), the initial layer thickness *h*_0_ and the contact angle *θ* between the polymer and the stamp. According to the concept of ‘partial cavity filling’ we assume that all the polymer available has been squeezed into the cavities and that a horizontal meniscus has formed between the stamp sidewalls, the target shape for lithography applications. With T-NIL, volume conservation holds in good approximation for a polymer above its glass transition, similar to the situation with (almost) liquid low viscosity resins used in UV-NIL. Furthermore, as all geometries are small, the effect of gravity can safely be neglected, and the horizontal meniscus formed within a cavity has a spherical or cylindrical shape.

This purely geometric guiding chart is already adequate to discuss the lower limit of the window for defect-free processing (at low mean filling levels $\bar{h}$), under practical, application-specific aspects. A correct identification of the upper limit (at high mean filling levels $\bar{h}$), will require a modification, namely the consideration of instabilities, as addressed in paragraph 5.

For simplicity we develop the guiding chart in the first instance by taking a frequently met example, the imprint of a linear, 1-dimensional grating with a certain duty cycle *s/w*. We assume the stamp to be patterned over its whole area, so that edge effects are avoided [34,38]. As an extension, the basic relationships for a guiding chart referring to a 2-dimensional grating are given in Appendix B.

### 4.1. Construction of the Geometric Guiding Chart

**Figure 3:**
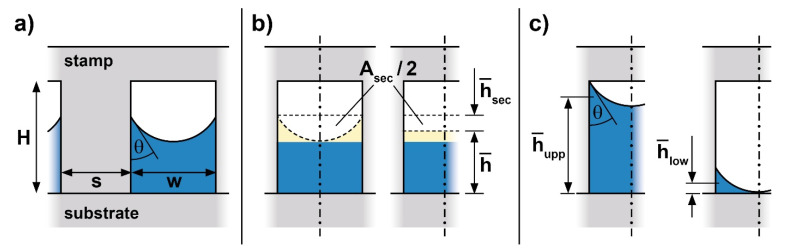
Definitions in view of the specification of a purely geometric processing window; cross-section through periodic linear structures. (**a**) Imprint situation: Stamp with geometries *s*, *w,* and *H*; within the partly filled cavities the polymer surface adopts a meniscus according to its contact angle *θ*; (**b**) partitioning of cross-sectional areas (see text); *A_sec_* refers to the circular section as indicated (mean filling height $\bar{h}$, mean height of circular section ${\bar{h}}_{sec}$). (**c**) Upper and lower boundaries: Polymer meniscus touches the ceiling of the stamp cavity at the corners (mean polymer height ${\bar{h}}_{upp}$) and the substrate in the center of the cavity (mean polymer height ${\bar{h}}_{low}$), respectively.

Figure 3 gives details of the quantities relevant to describe the structures obtained with a linear grating under ‘partial cavity filling’ conditions. Figure 3a illustrates the cross-sectional situation. The polymer available per period within the initial layer (Figure 1) is *h*_0_(*w* + *s*), which leads to a mean filling height $\bar{h}$ (see Figure 1 and Figure 3) in the periodic cavities of

(1) $$\bar{h}=h_{0}\left(\frac{w+s}{w}\right)=h_{0}\left(1+\frac{s}{w}\right)$$

**Figure 4:**
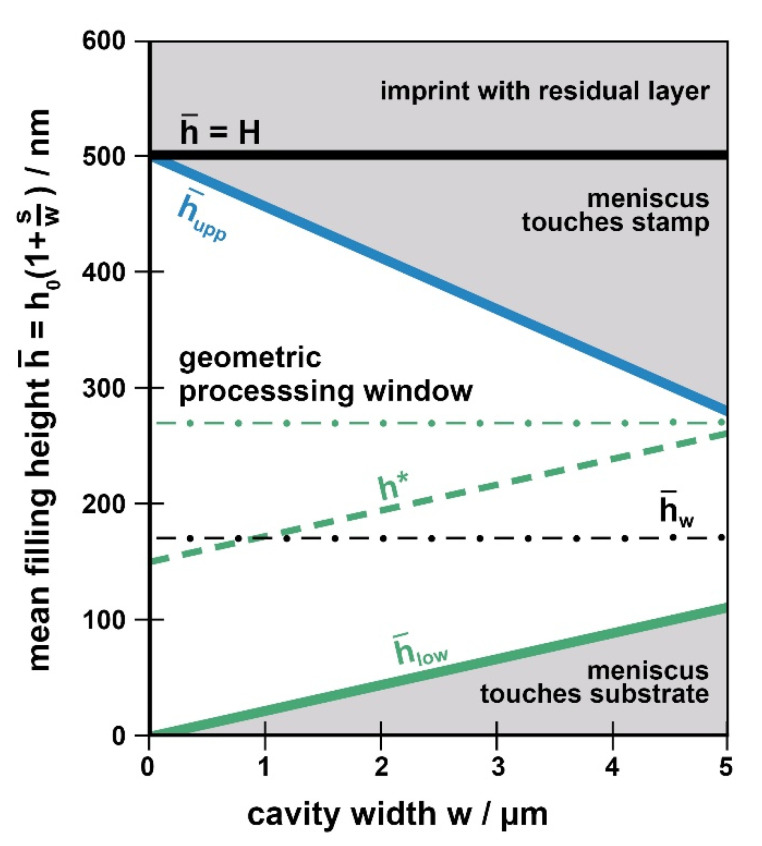
Example of a guiding chart for defect-free imprint with negligible residual layer, considering purely geometric limits (mean filling height $\bar{h}$ as a function of cavity width *w* for a contact angle of *θ* = 75° and a stamp height of *H* = 500 nm). The geometric processing window considering imprint-related issues (bright) is limited at its top and its bottom. The following boundaries and heights are indicated: $\bar{h}=H$ Exact filling of the stamp cavities without residual layer. ${\bar{h}}_{upp}$: Meniscus in stamp cavity reaches stamp ceiling at edges. ${\bar{h}}_{low}$: Centre of the meniscus touches substrate. ${\bar{h}}_{w}$: Optimum choice of mean filling height with mixed cavities (dash-dotted). h*: Additional boundary when a minimum polymer height is required (dashed, see text).

We identify this mean filling height as the parameter of interest as it is directly related to the initial layer thickness *h*_0_ to be chosen experimentally and the duty cycle (here *s*/*w*). Therefore, our guiding chart, Figure 4, maps the mean filling height, $\bar{h}$, for a range of cavity widths, *w*.

When this mean filling height $\bar{h}$ equals the stamp height *H* the cavities are completely filled without any residual layer. This is our first boundary for the guiding chart,

(2) $$\bar{h}=H;$$

it separates the regime with a residual layer remaining ($\bar{h}>H$) from the one of ‘partial cavity filling’ ($\bar{h}<H$), without residual layer.

Within the regime of ‘partial cavity filling’ two further geometric boundaries exist, as illustrated in Figure 3c, an upper one, where the meniscus just meets the edge of the stamp (mean filling height ${\bar{h}}_{upp}$), and a lower one, where the center of the meniscus touches the substrate (mean filling height ${\bar{h}}_{low}$).

Thanks to the cylindrical shape of the meniscus in our linear cavities the respective mean filling heights can easily be calculated based on the area of a circular section *A_sec_* as indicated in Figure 3b [45]. The mean height of such a circular section, ${\bar{h}}_{sec}$, when expressed by the parameters *θ* and *w* (the parameters involved here) amounts to

(3) $${\bar{h}}_{sec}=\frac{A_{sec}}{w}=\frac{\pi \;\left(1-\theta /90{}^{\circ}\right)-\sin2\theta }{{8\;\cos}^{2}\theta }\;\cdot \;w.$$

This relationship helps to determine the upper and lower boundaries, as depicted in Figure 3c. The upper limit (where the polymer meniscus touches the stamp ceiling at the corners of the cavity) is reached at a mean filling height of

(4) $${\bar{h}}_{upp}=H-{\bar{h}}_{sec}=H-m_{upp}\;\cdot \;w,$$

whereas the lower limit (where the polymer meniscus touches the substrate in the center of the cavity) is given by

(5) $${\bar{h}}_{low}=\frac{1-\sin\theta }{2\cos\theta }\cdot w-{\bar{h}}_{sec}=m_{low}\cdot w.$$

This lower limit is also reported by other groups [55].

These two geometric boundaries depend linearly on the cavity width *w*; the respective slopes *m_upp_* and *m_low_* are defined by the contact angle *θ* between the polymer and the stamp which, for a given imprint situation, is constant (characteristic values see Table 1).

**Figure 5:**
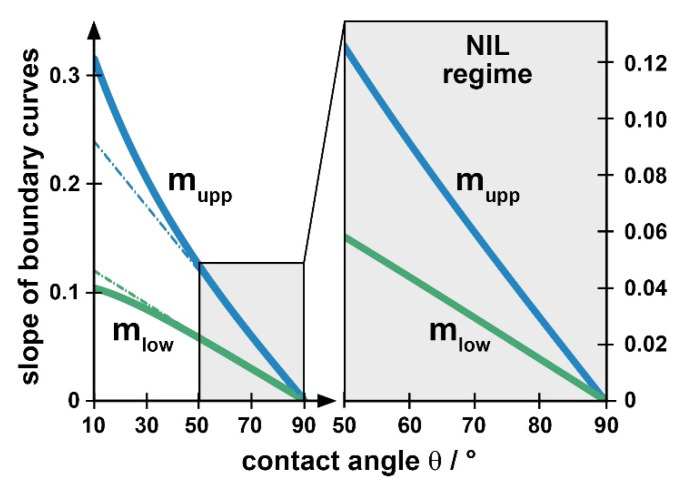
Non-dimensional slopes of the upper (blue) and lower (green) boundary for the formation of a horizontal meniscus in a linear cavity as a function of the contact angle *θ* of the imprinted polymer with respect to the stamp. With high contact angles the linear approximations are adequate (dash-dotted).

To simplify the construction of the guiding chart for users the slopes of the two boundaries, *m_upp_* and *m_low_* (Equations (4) and (5)), are plotted in Figure 5 as a function of the contact angle *θ*. Two linear approximations to these curves are indicated, representing the slopes with high contact angles, *θ* ≥ 50°. These approximations are well-suited for contact angles that are typical of nanoimprint (see Table 1), the non-linear part of the relationships being meaningless for practical applications. The approximations are given by the numerical relationship

(6) $$m_{upp}\approx 0.27-3\cdot 10^{-3}\frac{\theta }{\deg}\approx \;2\;m_{low}$$

and differ simply by a factor of 2. In most cases, Equation (6) is appropriate to determine the values for the two limiting slopes, as an alternative to reading the approximate value from Figure 5. 

The geometric guiding chart (mean filling height $\bar{h}$ over cavity width *w*) is constructed by choosing a range of cavity widths of interest and by indicating the height of the stamp to be used, *H*, as an upmost limit for $\bar{h}$. Then the two straight lines representing the upper and lower boundaries, ${\bar{h}}_{upp}$ and ${\bar{h}}_{low}$ are drawn, taking the slopes *m_upp_* and *m_low_* from Figure 5 or Equation (6). The region between ${\bar{h}}_{upp}$ and ${\bar{h}}_{low}$ is the available processing window, under purely geometric, imprint-related limitations. Obviously, the cavity width sets an absolute limit for nanoimprint under ‘partial cavity filling’ conditions for lithography purposes, with a specific height *H* of the stamp.

### 4.2. Discussion of the Geometric Guiding Ghart

Figure 4 gives an example of a preliminary, purely geometric guiding chart. As an example, we take a contact angle of *θ* = 75° and consider cavities *w* of up to 5 µm width. We assume a stamp of 500 nm in height, as indicated by the horizontal line at $\bar{h}=H$. The slopes *m_upp_* and *m_low_* from Figure 5 (*m_upp_* ≈ 0.045 = 45 nm/µm = 2 *m_low_*) are used to draw the boundaries ${\bar{h}}_{upp}$ and ${\bar{h}}_{low}$. Note that precision of the values is not an issue for constructing the guiding chart in praxis; the intention behind it is just to become familiar with the limitations existing with ‘partial cavity filling’. Any choice of initial layer thickness too near to any limit does not make sense in praxis, as a lot of the parameters required for constructing the guiding chart are known as approximate values only. In addition, small local variations of the geometries (e.g., stamp roughness) may lead to a random fluctuation of the boundaries.

#### 4.2.1. Imprint-Specific Issues

Only within the region between ${\bar{h}}_{upp}$ (blue line) and ${\bar{h}}_{low}$ (green line) a vanishing residual layer together with a horizontal meniscus in the cavities can be obtained. With increasing cavity width, the window of allowable mean filling heights $\bar{h}$ narrows as the risk that the meniscus touches the substrate or the ceiling of the stamp cavity increases. For any cavity width, the range of allowed initial layer thicknesses *h*_0_ (corresponding to $\bar{h}$) can easily be derived from the stamp duty cycle according to Equation (1).

Generally, the wider the cavities of a linear grating (at a duty cycle *s/w* = const) the more accurate the initial layer *h*_0_ has to be chosen to avoid defects. With varying cavities, the ‘most save’ initial layer is the one resulting in a mean filling height of ${\bar{h}}_{w}\approx H/3$, due to the relationship of Equation (6); it provides defect-free imprint with vanishing residual height over the widest range of cavity widths.

#### 4.2.2. Application-Specific Issues

So far, imprint-specific issues were addressed. Of course, choosing the initial layer with the help of the guiding chart cannot be based on imprint-related issues only; however, application-specific issues have to be considered, too. Typically, with NIL as a lithography process, a mask shall be provided for subsequent etching. When an intermediate lift-off process is intended (e.g., to invert the tone) the main issue is that the polymeric structures within the cavities are without holes. Then, theoretically, the processing window already discussed is usable, with a lower limit ${\bar{h}}_{low}$. However, to be on the safe side and to ensure that the substrate is masked even with local non-uniformities it is advised to prescribe a certain minimal polymer height, h*, in praxis. Moreover, when the imprinted structures shall provide the etching mask directly, this lower limit is defined by the etching process. As dry etch selectivity is limited, the minimum polymer height required to mask the substrate depends on the dry etching process. Its value is known by the user, only; any generalizing assumption is not possible. However, with h* at hand this additional limit can be integrated into the guiding chart. As ${\bar{h}}_{low}$ indicates a vanishing polymer height in the center of the cavity, this line has to be shifted upwards by h*. This additional practical boundary further narrows the suitable processing window from its bottom. As an example, a safety margin (or etch-induced limit) is indicated in Figure 4 assuming an arbitrary value of h* = 150 nm (which would for example not be reached with the example in Figure 2(b1), despite the continuous meniscus). With this example cavities of *w* ≥ 5 µm are critical. 

Combining the processing window for defect-free imprint with this additional, application-related boundary clearly indicates, up to which cavity size partial cavity filling is suitable with periodic, linear structures to provide a polymeric mask for the specific dry etching process to be used. As a consequence, under such conditions the regime of higher filling levels, $\bar{h}$ > h*, is of primary practical interest within the defect-free window. Of course, the filling height providing the best choice for a wide range of cavities then shifts upwards, accordingly (h*).

#### 4.2.3. Applicability

In the present form, the guiding chart, Figure 4, is applicable when just geometric factors represent the limits. This is the case when imprinting materials of very high viscosity, in the range of *η* ≈ 10^6^ Pas, as it would apply to T-NIL at a very low temperature. With lower viscosities, *η* ≤ 10^4^ Pas (a more typical processing regime with T-NIL), the polymer layer may suffer from instabilities developing within characteristic processing times, resulting in additional masking defects. UV-NIL may be even more prone to instabilities, due to the typically low viscosities. These instabilities are addressed in the next paragraph.

## 5. Processing Window with Instabilities

**Figure 6:**
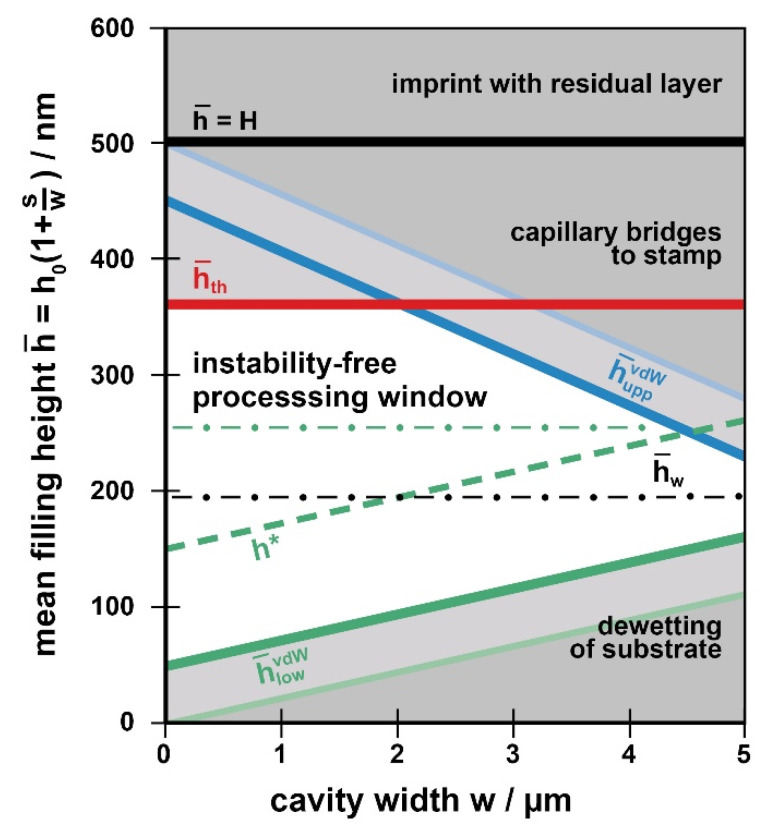
Example of a modified guiding chart for defect-free imprint with negligible residual layer similar to Figure 4, but additionally considering instabilities. The processing window (bright) is further reduced by boundaries indicating instabilities, namely ${\bar{h}}_{upp}^{vdW}$ and $\bar{h}{\;}_{low}^{vdW}$: Instabilities due to van der Waals forces. ${\bar{h}}_{th}$: Instabilities due to temperature differences (with instabilities due to electrostatic forces ${\bar{h}}_{th}$ would be replaced by ${\bar{h}}_{el}$).

Before revising the geometric processing window with respect to instabilities a basic view of the common knowledge is provided here. As our focus is on a practical manageability, the complex relationships existing with instabilities are generalized and presented by means of diagrams enabling the user to exploit such knowledge for his own specific processing situation. To explain the origin of the limitations induced by instabilities the physical background is summarized in short; an understanding of this background is not required, neither for constructing nor for using the guiding chart to identify the defect-free processing window in praxis. Readers interested primarily in the use of the guiding chart may skip part 5 (5.1 refers to the general physics underlying, and part 5.2 refers to the specific parameters entering the guiding chart). For the construction of the guiding chart, just the results will be used, namely Figures 6 and 8, together with the definition of the respective process parameters of relevance, Equations (10), (12) and (14).

### 5.1. Physical Background

Basically, the surface of a polymeric liquid, when near an interface, is subjected to interaction with this interface. With a thin film it simply is the interaction with the substrate, which may lead to a de-wetting of the film [68]. In the case of nanoimprint, the situation is more complex, as there may be an interaction that exists with the substrate, in addition to the stamp [69]; this is the situation of the polymer within a cavity under ‘partial cavity filling’ conditions. In particular, when an instability due to interaction with the stamp occurs, a small undulation of the polymer height will grow. When the stamp ceiling is reached somewhere after some time, this local stamp contact results in a reconfiguration of the polymer within the cavity to minimize the free energy, the faster the lower the viscosity. Such a reconfiguration may lead to a de-wetting between the contact points. Of course, a de-wetting to the substrate is also possible without stamp contact; then the interaction with the substrate is dominating as characteristic of very thin layers. Typically, a ‘linear stability analysis’ [52,70,71,72] is performed to identify those undulations that grow fastest, the only ones to be observed in the experiment.

#### 5.1.1. Driving Forces with NIL

Driving forces for instabilities in general are numerous [73]. With nanoimprint, the most important drivers are addressed by Schäffer [71,72,74,75]—our treatment is based on his work. These drivers are van der Waals forces, forces due to temperature gradients and electrostatic forces. Van der Waals forces exist in any imprint configuration; temperature gradients are specific of non-isothermal processes; electrostatic instabilities require the application of an external voltage. 

Independent from the imprint technique used, T-NIL or UV-NIL, van der Waals forces are always active to drive instabilities, in particular with thin fluid films [76]. With T-NIL, there will be instabilities from temperature gradients as well. Temperature gradients are most obvious with imprint systems featuring single-sided heating [77,78,79]. However, systems serving for iso-thermal processing also often feature temperature gradients that are less obvious. With parallel-plate systems, temperature differences of 1–2 °C are typical during imprint. Though small, we found that such temperature differences may already induce instabilities [45]. Furthermore, during the heat-up and cooling phase temperature differences between the heating plates may be even larger; 10 °C are not unusual [46]. At temperatures above the glass transition of the imprint polymer this heating/cooling phase has to be included when considering instabilities with T-NIL. We anticipate that with T-NIL, barely any system exists where instabilities from temperature gradients can safely be ignored. The system used for our experiments features a temperature gradient of about 10 °C during heat-up and a temperature gradient of 1–2 °C during imprint [46]. The temperature gradients with T-NIL are system-specific.

Electrostatic forces may also be system-specific; this is the case when they serve to provide the pressure for imprinting [80]; typically, this works with curable materials of low viscosity at room temperature as generally used with UV-NIL; we will refer to this technique as ‘el-UV-NIL’. In this case, the existence of electrostatic forces is obvious; however, due to their coupling with the imprint pressure a free choice of their size is impeded. Furthermore, electric fields may be applied intentionally during T-NIL to induce certain physical effects, e.g., the phase separation of a block copolymer [81,82]. Then both, electrostatic and thermal instabilities may occur, the largest one dominating the situation; we will refer to this as ‘el-T-NIL’. Moreover, electric fields may also exist unintentionally, e.g., when T-NIL is performed via current-induced heating of the stamp itself [77,78]; whether or not electric fields are present in such a case depends on the implementation of the electrodes and their grounding situation. Such unintended co-action of temperature differences and electric field ranks again as ‘el-T-NIL’. 

To address all these aspects with NIL, our analysis will cover van der Waals forces, temperature gradients, and electrostatic forces.

#### 5.1.2. Stability Analysis with NIL

To identify the basic relationships, we follow the conventional procedure and perform a linear stability analysis. Here it refers to a polymeric layer of mean height $\bar{h}$ within a stamp cavity of height *H*, according to Figure 1(b2) and Figure 3b. The following simplifications are made. (i) We separate the imprint from the stability analysis; thus, we assume the instabilities to develop when the polymer has already been squeezed into the cavities. (ii) We assume that an equilibrium is reached within the processing time. (iii) We ignore the lateral meniscus in the cavity and work with the mean filling level $\bar{h}$ instead. (iv) We consider instabilities in vertical direction only; due to some randomness in the experimental conditions (e.g., stamp roughness [83]), an exact consideration of lateral boundaries is less meaningful. 

We found that this simplified analysis, in combination with the geometric analysis already discussed, is appropriate to understand all typical phenomena observed experimentally with instabilities during nanoimprint. In particular, we make use of the basically periodic nature of the instability phenomenon to apply it to the lateral cavities. With linear cavities of a few micron width, the experiments (see Figure 2(b2)) indicate that instabilities start from the sidewalls of the stamp and then develop in the third dimension, along the length of the respective cavity, during the reconfiguration phase.

Furthermore, as our aim is to draw practical conclusions from such a stability analysis for nanoimprint, we restrict the presentation here to those equations/correlations that are urgently required to explicate the actual proceeding. Details of the analysis are given in Ref. [45] and in parts in Ref. [46]. To assign the equations given in Ref. [46] to the actual situation the initial layer thickness *h*_0_ and the gap height *d* (distance between polymer surface and stamp ceiling) have to be replaced by the respective actual quantities, the mean filling height in the cavities, $\bar{h}$, and the height of the stamp structures, *H*. 

Mathematical treatment starts from a polymer of mean height $\bar{h}$ located between the substrate and the stamp (at a distance *H*). Its surface, the polymer/air-interface, is subjected to interactions with other nearby interfaces, the polymer/substrate and the air/stamp-interface. These interactions are characterized by a respective pressure, *p*, which may ‘stabilize’ or ‘destabilize’ the layer. Destabilizing pressures (inducing instabilities by amplifying fluctuations) result from the forces identified as drivers, van der Waals forces (*p_vdW_*, between the polymer surface and the stamp or substrate), thermal forces (*p_th_*, when a temperature gradient exists between stamp and substrate), and electrostatic forces (*p_el_*, when a potential difference *U* is effective between stamp and substrate). These ‘destabilizing’ pressures (*p_des_* = *p_vdW_* + *p_el_* + *p_th_*) are counteracted by the surface tension of the viscous layer via the Laplace pressure *p_La_* (that tries to hold the polymer-air interface as flat (and thus, as small) as possible to minimize the respective energy); thus, *p_La_* acts as the major ‘stabilizing’ pressure (*p_La_* = *p_stab_*) that damps instabilities.

With imprint, the polymer volume remains conserved, with T-NIL above the glass transition of the polymer and with low viscosity liquid resins used for UV-NIL as well. Therefore, under the action of pressures the viscous polymer layer is described by the continuity equation, here a differential equation for the polymer height *h*(*x,t*). With our 2D-problem (linear grating), solutions for this differential equation are harmonic in lateral direction *x* (with wave numbers *k_i_*) and exponential in time *t* (with time constants *τ_i_*). With one of these harmonic solutions (i) the fluctuating polymer height follows

(7) $$h_{i}\left(x,t\right)\;=\;\bar{h}\;\left(1+\;\delta \cdot \cos\left(\;\frac{2\pi x}{k_{i}}\;\right)\exp\left(\;\frac{t}{\tau_{i}}\;\right)\right)\;,$$

with δ some small quantity (δ << 1) defining the undulation of the initial amplitude of the polymer height over its mean value, $\bar{h}$. 

As just the fastest growing undulation shows up in the experiments, the solution with the smallest time constant is the only one of practical interest. Based on Equations (3) and (4) of ref [46] this smallest time constant, *τ*, is defined by the mean layer thickness $\bar{h}$ and the change of the destabilizing pressure with layer thickness, according to

(8) $$\frac{1}{\tau }=\frac{{\bar{h}}^{3}}{{12\eta }_{p}\gamma_{p}}\;{\left[{\left(\frac{\partial p_{\mathrm{des}}}{\partial h}\right)}^{2}\right]}_{h=\bar{h}},$$

with *η_p_* and *γ_p_* the viscosity and the surface tension of the polymer under processing conditions.

When, after a certain interaction time *t_p_* (characteristic for the process), the maximum of the fastest growing undulation reaches the ceiling of the stamp cavity ($h_{\max}\left(t_{p},\tau \right)=H$) a polymeric bridge forms. Combining Equations (7) and (8) under such conditions results in the relationship

(9) $$0\;=\;P_{0}\;\cdot \;\frac{{\bar{h}}^{3}}{12}\;\cdot {\left[{\left(\frac{\partial p_{\mathrm{des}}}{\partial h}\right)}^{2}\right]}_{h=\bar{h}}-\;\ln\left(\frac{H-\bar{h}}{\delta \cdot \bar{h}}\right),$$

with

(10) $$P_{0}\;=\;\frac{t_{p}}{\eta_{p}\gamma_{p}}.$$

*P*_0_ is a basic processing parameter. The quantity dominating its size is the viscosity *η_p_*, which may vary over six decades with the different imprint techniques. It will be used later on to define a process-specific parameter that is characteristic of thermal and electrostatic instabilities.

Equation (9) is an implicit relationship between the mean polymer height in the cavity, $\bar{h}$, and a given stamp height, *H*, with any destabilizing pressure of interest; the basic processing parameter *P*_0_ being constant with a specific imprint situation. A solution $\bar{h}\;\left(H\right)$ implies that the right-hand side of Equation (9) equals zero; then the polymer level $\bar{h}$ is high enough that the undulations just touch the stamp within the interaction time, bridging the initial gap, ($H-\bar{h}$). This is the boundary looked for that separates the defect-free from the defective regime. A choice of $\bar{h}$ below the boundary does not result in instabilities, but beyond it does.

Calculating the boundary means to find a solution $\bar{h}\;\left(H\right)$ of Equation (9). Generally, the destabilizing pressure of interest is inserted in Equation (9) and the implicit mathematical relationship is solved for its null for pairs of variates of ($\bar{h},H$), taking the specific material and process parameters into account as explained below with thermal and electrostatic instabilities.

### 5.2. Modification of the Guiding Chart with Instabilities

In the following, the three destabilizing forces (van der Waals, thermal, electrostatic) are each addressed to identify the respective boundaries that further limit the processing window with instabilities, as exemplified in the modified guiding chart, Figure 6. Its construction is based on the limits already discussed with the purely geometric processing window, Figure 4.

To provide practical access to this complex physical regime we will generalize the results as far as possible. This is facilitated by the fact that within the typical parameter range the material properties (dielectric, thermal) are not crucial for the main findings. With thermal and electrostatic instabilities, we will identify a single process-specific parameter (*P_th_, P_el_*), combining the basic processing parameter *P*_0_ (Equation (10)) with the external control parameter applying, the temperature difference Δ*T* or the voltage applied *U*. The solutions of the problem are presented as a graph. The limiting polymer height can be read from the graph with this single process-specific parameter at hand, for any stamp height.

#### 5.2.1. Van der Waals Forces

Van der Waals forces between plane interfaces are proportional to the reciprocal of the respective distance to a power of three; accordingly they affect thin layers or narrow gaps only. The material characteristics enter the relationship via the respective Hamaker constant being dielectric in nature [76,84]. The situation under typical imprint conditions is addressed in detail in Refs. [45,46]. Van der Waals forces cannot be controlled by an external processing parameter, they are always present; it is fully adequate to make use of a generalized result in praxis.

It was found that van der Waals forces lead to a wetting of the stamp ceiling with gaps of $H-\bar{h}\leq 50\;\mathrm{nm}$; similarly, de-wetting at the substrate occurs with layers of $\bar{h}\leq 50\;\mathrm{nm}$. With respect to the guiding chart, it simply means that the upper and the lower boundaries (according to Equations (4) and (5)) have to be shifted by 50 nm downwards and upwards, respectively. This is exemplified in Figure 6 by the lines ${\bar{h}}_{upp}^{vdW}$ and ${\bar{h}}_{low}^{vdW}$. The value of 50 nm may be seen as a worst-case limit; it results from a viscosity low enough and an interaction time long enough (namely the size of the parameter *P*_0_); with T-NIL at a viscosity of 10^5^ Pas it may be somewhat smaller than 50 nm. As already mentioned in context with Figure 4, at viscosities of ≈10^6^ Pas instabilities are not relevant, independent from their source.

#### 5.2.2. Temperature Gradients

The effect of temperature gradients depends on an external control parameter, the size of Δ*T*, the temperature difference between the surfaces of the stamp, and the substrate with T-NIL. Defect formation is independent from the direction of the temperature gradient [72,74]. As Δ*T* is not known a priori but system-specific, the respective boundary has to be determined from Equation (9), following the general procedure (vanishing of the right-hand side of the equation). The pressure derivative with temperature gradients reads [45,46]

(11) $${\left(\frac{\partial p_{th}}{\partial h}\right)}_{h=\bar{h}}=\;C_{th}\cdot \;{\left[\kappa_{air}\bar{h}+\kappa_{pol}\left(H-\bar{h}\right)\right]}^{-2}\;\cdot \;\Delta T,$$

with *κ_air_* and *κ_pol_* the thermal conductivities of the air gap and the polymer, respectively. The first term of this product is a constant (*C_th_*) summarizing thermal material parameters only. The second term combines material parameters and geometries in a way that is typical of temperature gradients. The third term is the external control parameter Δ*T*. Together with the parameter *P*_0_ already introduced (Equation (9)) the latter defines the process-specific parameter

(12) $$P_{th}=P_{0}\cdot {\Delta T}^{2}.$$

**Figure 7:**
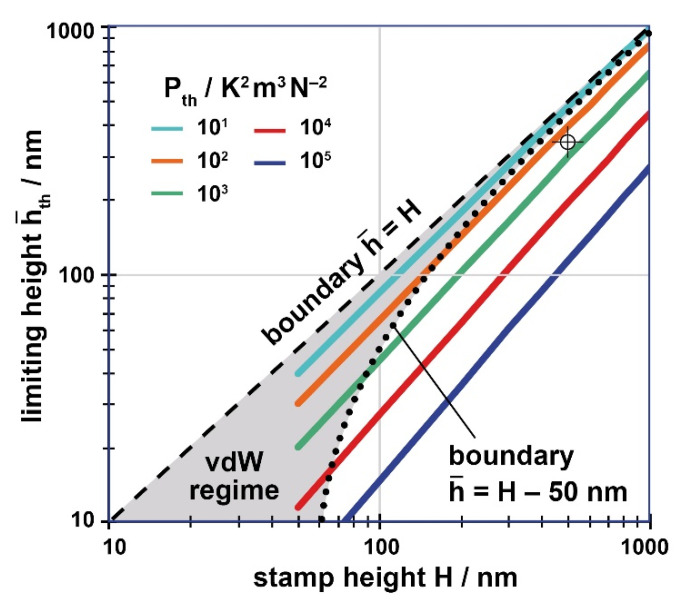
Instabilities due to temperature differences, Δ*T*. Limiting values of mean polymer height in the cavity, ${\bar{h}}_{th}$, as a function of the stamp height, *H*, from which thermal instabilities clip the processing window for defect-free imprint with negligible residual layer. The process-specific parameter *P_th_* [K^2^m^3^/N^2^] is varied. (Material parameters used [45,72,75]; *κ_air_* = 0.034 W/Km, *κ_pol_* = 0.16 W/Km, *C_th_* ≈ −5 × 10^−6^ (W/K)^3.^s/m^4^ based on an effective sound velocity of 1850 m/s). The ‘cross’ refers to the example used for discussion with *P_th_* ≈ 300 K^2^m^3^/N^2^, see text.

The size of the parameter *P_th_* has to be determined by the user. Solutions of Equation (9), regarding the characteristic relationship of Equation (11) can be determined by just considering the value of *P_th_*, as ${\bar{h}}_{th}=\bar{h}\left({H,\;P}_{th}\right)$. The respective limiting height ${\bar{h}}_{th}$ is displayed in Figure 7 for values of 10^1^ ≤ *P_th_*/K^2^m^3^N^−2^ ≤ 10^5^. Of course, any polymer height $\bar{h}$ will be smaller than the stamp height H, so that all solutions lie below $\bar{h}=H$ (dashed line in Figure 7).

With respect to the guiding chart (Figure 6), instabilities induced by temperature gradients represent a supplementary boundary limiting the window for defect-free imprint with vanishing residual layer from its top, with small cavity widths. To add this boundary the parameter *P_th_* has to be determined from Δ*T* (maximum or characteristic value), *t_p_* (characteristic time with Δ*T*), *η_p_* and *γ_p_*, the viscosity and the surface tension of the polymer under processing conditions, here at the respective temperature. 

With the value of P_th_ (in units of K^2^m^3^/N^2^) the respective maximum height ${\bar{h}}_{th}$ can be read (and interpolated, if necessary) from Figure 7 and can be drawn as a horizontal line into the guiding chart, Figure 6 (for reasons see Appendix A). The lower ${\bar{h}}_{th}$, the more the defect-free processing window is clipped at its top. As we found that typically one of the instabilities dominates [46], just the lowest upper limit is effective (min{${\bar{h}}_{th},\;\bar{h}{\;}_{upp}^{vdW}$}). To indicate which limit is dominating, Figure 7 also contains the van der Waals boundary, $\bar{h}={\bar{h}}_{upp}^{vdW}=H-50\;\mathrm{nm}$ (dotted). Thus, only with a process-specific parameter beyond *P_th_* ≈ 10^2^ K^2^m^3^/N^2^ temperature gradients exceed van der Waals forces and are effective to decrease the defect-free processing window.

#### 5.2.3. Electrostatic Forces

The effect of electrostatic forces depends on an external parameter too, the voltage *U* applied between stamp and substrate (electrically isolated from each other) [70,71,72,85,86,87]. The procedure is parallel to the previous one with a temperature gradient. Here the pressure derivative reads [45,46]

(13) $${\left(\frac{\partial p_{el}}{\partial h}\right)}_{h=\bar{h}}=\;C_{el}\cdot \;{\left[\varepsilon_{air}\bar{h}+\varepsilon_{pol}\left(H-\bar{h}\right)\right]}^{-3}\;\cdot \;U^{2},$$

with $\varepsilon_{air}=\varepsilon_{0}$ and $\varepsilon_{pol}=\varepsilon_{0}\varepsilon_{p}$ ($\varepsilon_{0}$ the dielectric constant) for the air gap and the polymer, respectively. Again, the first term, *C_el_*, summarizes material parameters only, now dielectric ones. The second term combines material parameters and geometry parameters in a way that is typical of electrostatic forces (note the exponent differing from Equation (11)) and the third term is the external control parameter, the voltage, entering here as *U*^2^. Similar to the previous case the situation is characterized by a process-specific parameter, now

(14) $$P_{el}=P_{0}\cdot U^{4}$$

**Figure 8:**
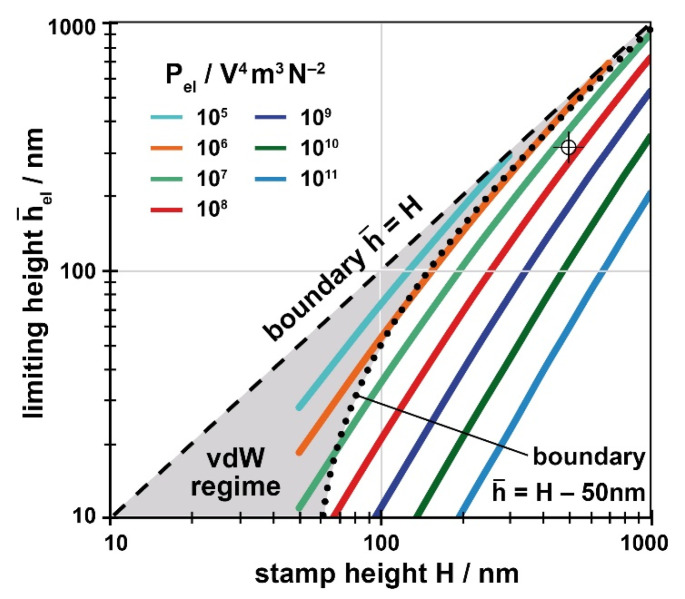
Instabilities due to electrostatic forces (potential difference *U*). Limiting values of mean polymer height in the cavity, ${\bar{h}}_{el}$, as a function of the stamp height, *H*, from which on electrostatic instabilities clip the processing window for defect-free imprint with negligible residual layer. The process-specific parameter *P_el_* [V^4^m^3^/N^2^] is varied. (Material parameter used; $\varepsilon_{p}$ = 3, *C_el_* ≈ −1 × 10^−10^ As/Vm). The ‘cross’ refers to the example chosen for discussion with *P* ≈ 3 × 10^7^ V^4^m^3^/N^2^, see text.

The solutions of Equation (9) regarding Equation (13) are plotted in Figure 8; of course, it still holds that $\bar{h}\leq H$. Again, the process-specific parameter *P_el_* has to be determined by the user (units now V^4^m^3^/N^2^) and a limiting value ${\bar{h}}_{el}$ for the stamp used (height *H*) has to be read from Figure 8 and implemented in Figure 6, again a horizontal line at ${\bar{h}}_{el}\;=\bar{h}\left({H,P}_{el}\right)$. Only with *P_el_* > 10^6^ V^4^m^3^/N^2^ electrostatic instabilities have to be considered in praxis, as evident from Figure 8; with smaller values of *P_el_* they are dominated by the van der Waals instabilities always present. 

## 6. Working with the Guiding Chart

Though the idea behind the guiding chart is to simplify the use of ‘partial cavity filling’ with NIL by generalization (in particular the adequate choice of the initial layer thickness *h*_0_), every user has to construct his own guiding chart, being specific for his imprint situation and the imprint system used. The application in mind for nanoimprint as a lithography process has to be included too (lift-off, direct dry etching).

### 6.1. Construction of a Specific Guiding Chart

For practical use, a specific guiding chart has to consider (i) geometric limitations, (ii) limitations induced by instabilities (with the exception of very high viscosities, ≈10^6^ Pas), and (iii) application-specific limitations. To construct the guiding chart the following quantities are required as input parameters.

- Stamp geometries. Height *H*, cavity width *w* of interest, duty cycle *s/w* (see Figure 1).
- Polymer data under processing conditions. Contact angle *θ* to the stamp, viscosity *η_p_*, surface tension *γ_p_* (estimates may be taken from Table 1).
- System data. Characteristic temperature difference Δ*T* and/or characteristic voltage *U*, as well as corresponding interaction time *t_p_*. Please note, Δ*T* and *U* refer to the values between the surfaces of substrate and stamp ceiling; these may differ from overall values (e.g., available from data log-files of the imprint system used), depending on the imprint configuration, e.g., thermal/electrical isolation. An estimate may be required.
- Application-related data. Minimum polymer height h* provided/required for lift-off/etching, with h* > 50 nm (van der Waals limit).

With these data at hand, the construction of the specific guiding chart exploits Figure 5 (alternatively Equation (6)) to find the geometrical limits. To identify the limiting heights induced by thermal or electrostatic instabilities, namely *h_th_* and *h_el_*, the relationships derived in chapter 5 are required. As already stated there, for a straightforward use, the results are presented in graphical form, Figure 7 referring to thermal instabilities and Figure 8 referring to electrostatic instabilities. To read the limiting height (${\bar{h}}_{th}$ or ${\bar{h}}_{el}$) the value of the respective process-specific parameter, *P_th_* or *P_el_*, has to be determined from the input parameters, namely $P_{th}=P_{0}\cdot {\Delta T}^{2}$ (Equation (12)) and/or $P_{el}=P_{0}\cdot U^{4}$ (Equation (14)), with $P_{0}=t_{p}/\eta_{p}\gamma_{p}$ (Equation (10)). The specific guiding chart will look similar to Figure 6, however adapted to the characteristic situation of the user.

The construction of the specific guiding chart then proceeds as follows:

1. Draw overall regime. Height $\bar{h}=H$, width about 3–5 times the cavity width *w* of interest.
2. Add geometric boundaries. Determine *m_upp_* and *m_low_* from Figure 5 or Equation (6) for the contact angle *θ* applying. Draw upper boundary ${\bar{h}}_{upp}$ as a straight line starting at $\bar{h}=H$ at *w* = 0, with a negative slope of *m_upp_*; draw ${\bar{h}}_{low}$ with the positive slope *m_low_*, starting from $\bar{h}\;=\;0$ at *w* = 0.
3. Indicate van der Waals limits. Draw ${\bar{h}}_{upp}^{vdW}$ and ${\bar{h}}_{low}^{vdW}$ as a parallel to ${\bar{h}}_{upp}$ and ${\bar{h}}_{low}$, shifted by 50 nm downwards and upwards, respectively.
4. Add minimum polymer height provided/required for application as a parallel to ${\bar{h}}_{low}$, shifted upwards by h*.
5. In case of T-NIL (or electrostatic NIL), add additional upper limit ${\bar{h}}_{th}$ (or ${\bar{h}}_{el}$) as a horizontal line; if both apply, take the smaller value. The respective height is read from Figure 7 (or Figure 8) taking the stamp height *H* and the value of the process-specific parameter applying, *P_th_* (or *P_el_*).

The processing window (range of values $\bar{h}$ for any cavity width *w*) available for using partial cavity filling for residual layer-free and defect-free imprint is the regime between the dominating lower limit, $\max\left\{h*,\;\bar{h}{\;}_{low}^{vdW}\right\}$, and the dominating upper limit, $\min\left\{{\bar{h}}_{th},\;\bar{h}{\;}_{upp}^{vdW}\right\}$, in analogy to Figure 6.

### 6.2. Discussion and Conclusions

For a general discussion, we take the guiding chart of our example, Figure 6. The geometries are similar to Figure 4, a stamp of height *H* = 500 nm and a range of cavity widths up to *w* = 5 µm. With thermal and electrostatic instabilities, we address a medium range of the external parameters, Δ*T* ≈ 10 °C and *U* ≈ 10 V. The respective interaction time *t_p_* for the development of the instabilities matches a specific imprint situation. Typical material parameters with T-NIL refer to Table 1; with UV-NIL the viscosities are in the range of 10^−1^–10^2^ Pas. The choice of the initial layer (see Equation (1), $\bar{h}=h_{0}\left(1+s/w\right)$) is exemplified by taking cavities of 1 µm width.

From this general discussion and the example with *w* = 1 µm the user may easily draw the conclusions for his own, specific situation, namely an adequate choice of the initial layer height *h*_0_. Furthermore, he may decide which measures are appropriate under his specific conditions/limitations to enlarge the processing window, so that ‘partial cavity filling’ can be used to imprint with a negligible residual layer, however, without defects.

#### 6.2.1. Lower Limit

At low filling levels, van der Waals forces are the only ones inducing instabilities. In the regime between $\bar{h}{\;}_{low}^{vdW}$ and ${\bar{h}}_{low}$ the risk is high that the polymer de-wets on the substrate. With lithography, this will result in mask defects, e.g., holes in the patterned polymer layer. Such structures are definitely unusable for lithography purposes, independent from the strategy followed (lift-off, direct etching). In praxis, there will always be some minimum polymer height h* required; with lift-off it may be some safety margin; when direct use of the polymeric structures as a mask for dry etching is intended, h* is determined from the selectivity of the dry etching process applied (as discussed in detail with the geometric processing window, Figure 4). Similar to the purely geometric guiding chart the lower boundary, $\bar{h}{\;}_{low}^{vdW}$, is mainly of academic interest; it becomes overruled and will be replaced by h* in praxis, with typically h* > $\bar{h}{\;}_{low}^{vdW}=\bar{h}{\;}_{low}+50$ nm.

#### 6.2.2. Upper Limit

At high filling levels, $\bar{h}$ is limited by van der Waals forces as well as thermal and electrostatic forces; as in general the lowest limit is dominating, the upper boundary is given by $\min\left\{{\bar{h}}_{th},\;{\bar{h}}_{el},\;\bar{h}{\;}_{upp}^{vdW}\right\}$ in general. The instabilities defining the upper boundary of the processing window (and thus the choice of the initial layer) depend on the specific technique applied with NIL.

***UV-NIL***: With UV-NIL neither temperature gradients nor electrostatic fields are present; therefore, just van der Waals forces may cause instabilities. Due to the low viscosity, the van der Waals limit of 50 nm is fully exploited. The upper boundary of the processing window is given by $\bar{h}{\;}_{upp}^{vdW}$ in Figure 6 (${\bar{h}}_{th}$ does not exist), the lower one by h* (or $\bar{h}{\;}_{low}^{vdW}$).

In the regime between $\bar{h}{\;}_{upp}^{vdW}$ and ${\bar{h}}_{upp}$, there is some risk that polymer bridges to the stamp ceiling develop. As long as their period is small and the polymer height remaining between the bridges is high, this is not critical (whilst h* is met); an example is given in Figure 2(c1). However, generally there is some risk of de-wetting between the bridges. This is most probable with wide cavities. As already addressed, local bridges lead to a rearrangement of the polymer, enhancing the risk of a local de-wetting of the substrate along the linear cavities, in the third dimension (see Figure 2(a3–5,b3,4)). The rearrangement process is a question of time and viscosity (and thus *P*_0_). To avoid instabilities safely, the regime between $\bar{h}{\;}_{upp}^{vdW}$ and ${\bar{h}}_{upp}$ is off-limits with UV-NIL, due to the low viscosity.

As the processing window is not further clipped at its top, it is wide, in particular with small cavities. However, due to the low viscosities and thus a high risk of rearrangement at random flaws of the stamp, the direct neighborhood of the upper boundary should be avoided in praxis.

With 1 µm wide cavities a maximum mean filling height of $\bar{h}$ ≈ 350 nm should be fully adequate and would provide a good masking height for etching. With a duty cycle of the stamp of *s*:*w* = 1:1 (2:1) the respective initial height required to provide this filling level amounts to *h*_0_ ≈ 175 nm (120 nm).

***T-NIL***: With T-NIL the additional limiting height in the guiding chart is ${\bar{h}}_{th}$, as indicated in Figure 6, so that the upper boundary of the processing window is represented by $\min\left\{{\bar{h}}_{th},\;\bar{h}{\;}_{upp}^{vdW}\right\}$. Obviously thermal instabilities are particularly effective with small cavities (high filling levels) and clip the window from its top. Only with T-NIL at untypically low imprint temperatures (viscosities of ≈10^6^ Pas) any limitation due to instabilities does not apply (*P*_0_, *P_th_* very small). In that case, the upper boundary would simply be ${\bar{h}}_{upp}$. These conditions would result in the widest defect-free processing window possible.

As a typical example, we determined *P_th_* ≈ 300 K^2^m^3^/N^2^ for an imprint with our system at a high temperature, 200 °C, from the values Δ*T* ≈ 10 °C, *t_p_* ≈ 15 min (during heat-up and cool-down [46]), *η_p_* ≈ 10^4^ Pas and *γ_p_* ≈ 30 mJ/m^2^. This results in a limiting filling height of ${\bar{h}}_{th}$ ≈ 320 nm (see ‘cross’ in Figure 7). As the viscosity is typically high with T-NIL, random flaws of the stamp are less effective to cause a rearrangement of the polymer within the cavities, so that the remaining processing window may be fully exploited in praxis.

With 1 µm wide cavities, a mean filling height of $\bar{h}$ ≈ 300 nm should be well suited to avoid instabilities, still a good masking height for etching. With a duty cycle of the stamp of *s*:*w* = 1:1 (2:1), the respective initial height required to provide this filling level amounts to *h*_0_ ≈ 150 nm (100 nm). With non-uniformity of the initial layer a somewhat lower value should be chosen.

To illustrate the benefit of the guiding chart the practical examples shown in Figure 2 that were obtained with T-NIL are assigned to the different regions. With a1 we are in the regime of a residual layer, beyond $\bar{h}$ = *H*. With a2 we are within the safe processing window for imprint (Figure 6). The same holds for b1, however depending on the application used, the limit h* may not be satisfied. With a3–5 and b3,4 we are beyond ${\bar{h}}_{th}$, the instabilities visible are thermally induced. Lastly, b2 and c4 are beyond ${\bar{h}}_{upp}$, the meniscus touches the stamp (Figure 4).

***el-UV-NIL***: With electrostatic nanoimprint, the horizonal line at ${\bar{h}}_{th}$ is replaced by ${\bar{h}}_{el}$ in the guiding chart, Figure 6. Therefore, the upper boundary is represented by $\min\left\{{\bar{h}}_{el},\bar{h}{\;}_{upp}^{vdW}\right\}$; again, the window is clipped from its top at small cavity widths (high filling levels).

As a typical example we determined *P_el_* ≈ 3 × 10^7^ V^4^m^3^/N^2^, from the values *U* = 10 V, *t_P_* = 20 min, *γ_p_* ≈ 40 mJ/m^2^ and *η_p_* ≈ 10 Pas. This results in a limit of the filling height of ${\bar{h}}_{el}$ ≈ 320 nm (see ‘cross’ in Figure 8—by chance similar to T-NIL). Due to the low viscosity, again the direct neighborhood of this upper limit should be avoided.

With 1 µm wide cavities a mean filling height of $\bar{h}$ ≈ 270 nm should be adequate to avoid instabilities. With a duty cycle of the stamp of *s*:*w* = 1:1 (2:1) the respective initial height required to provide this filling level amounts to *h*_0_ ≈ 135 nm (90 nm). With these low initial layer thicknesses non-uniformity may be critical.

***el-T-NIL***: With both external control parameters, a temperature gradient and a voltage as well, the dominating limit is again the lower one. Accordingly, the upper boundary of the processing window is given by $\min\{{\bar{h}}_{el},\;{\bar{h}}_{th},\;\bar{h}{\;}_{upp}^{vdW}$}; again, the window is clipped from its top at small cavity widths (high filling levels).

As an example, we address a situation met in an earlier investigation, the electrically assisted phase separation of a block-copolymer during T-NIL under ‘partial cavity filling’ conditions [82]. With this investigation, the voltage drop between substrate and stamp ceiling was about 10 V and the material was treated for 1 h at 170 °C (*η* ≈ 10^5^ Pas, *γ_p_* ≈ 33 mJ/m^2^). These values result in a process-specific parameter *P_el_* ≈ 10^4^ V^4^m^3^/N^2^. With this low value (caused by the high viscosity) electrostatic instabilities will not occur (Figure 8: *P_el_* > 10^6^ V^4^m^3^/N^2^ to dominate). Accordingly, our experiments were not affected by electrostatic instabilities (see Figure 2(c2)). The processing window appropriate was the one with T-NIL; accordingly, thermal instabilities were observed at high temperature (see Figure 2(c3)). Here, any minimum height with respect to dry etching was not an issue.

#### 6.2.3. Hidden Control Parameters

When instabilities are considered, the processing window (Figure 6) may seem somewhat constricted, in particular when direct etching with the polymeric mask is intended (h* high). Nonetheless, the concept of ‘partial cavity filling’ is powerful to imprint with a negligible residual layer in praxis; yet a well-prepared experiment is asked. The discussion of the processing window available clearly indicated that the upper boundary is the critical one in praxis. Even so, two parameters that are of major impact to widen the processing window may have escaped attention. These somewhat ‘hidden’ external control parameters are related to the stamp used. 

A stamp of adequate height providing a low contact angle widens the processing window substantially (*H* high, *m_upp_* and *m_low_* small). In praxis, *H* may be fixed otherwise. Similarly, choice of the contact angle is restricted as the polymer to be used may be prescribed. However, the contact angle is controlled by the surface properties of the stamp, too. A low surface energy and thus excellent anti-sticking properties are a question of technological diligence with stamp preparation. This emphasizes the impact of a well-controlled and reproducible anti-sticking treatment [63] to limit instabilities.

Furthermore, with small cavities, the upper limit may be further raised when ${\bar{h}}_{th}$ or ${\bar{h}}_{el}$ is high. With T-NIL, this is the case when performed under iso-thermal conditions in an imprint system featuring minimum temperature gradients (${\bar{h}}_{th}$ high). With el-UV-NIL, a compromise may be required to provide the imprint pressure required, but without being affected by instabilities. 

Of course, the (at least approximate) knowledge of the parameters characterizing the imprint system and the imprint material is a prerequisite to fully exploit this concept.

#### 6.2.4. Typical Regimes with NIL

**Figure 9:**
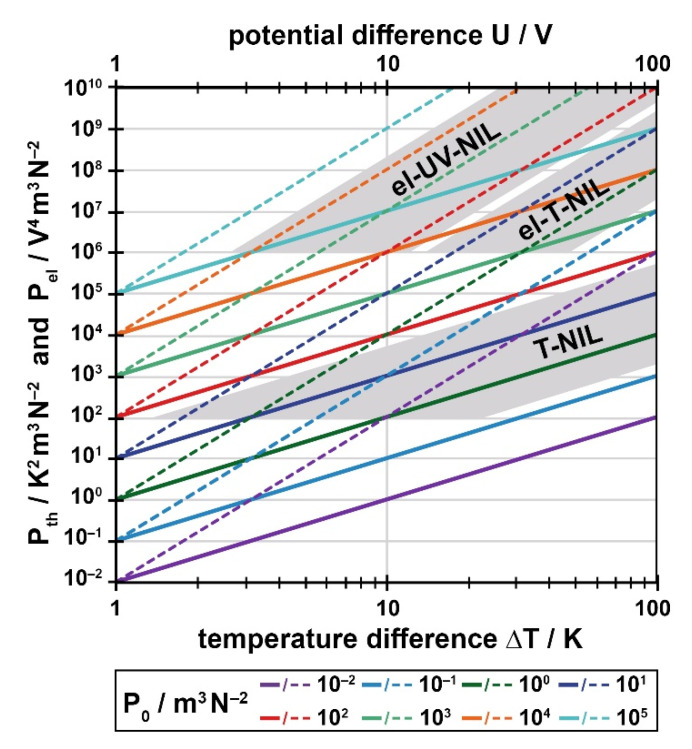
Role of the external control parameters Δ*T* and *U*. Process-specific parameters *P_th_* (full lines, lower axis) and *P_el_* (dashed lines, upper axis), with similar values of the basic processing parameter *P*_0_ (10^−2^–10^5^ m^3^/N^2^). The marked regions refer to typical parameter ranges with T-NIL, UV-NIL in an electrostatic imprint system (el-UV-NIL) and electrically assisted T-NIL (el-T-NIL), see text.

Finally, for practical purposes we illustrate the role of the external control parameters Δ*T* and *U* with instabilities. Therefore, regimes prone to instabilities will be indicated with thermal, electrostatic, and electrically assisted NIL. Again, a graphical representation is chosen, shown in Figure 9.

From Figure 7 and Figure 8, it is evident that the two process-specific parameters, *P_th_* and *P_el_*, are quite different in size (in parts due to Δ*T* and *U* entering by a power of 2 and 4, respectively). Both are compared to each other in Figure 9 to illustrate this difference, with similar values of P_0_. In accordance with typical imprint situations, the range of the external control parameters is chosen similarly, 1 °C ≤ Δ*T* ≤ 100 °C (lower axis) and 1 V ≤ *U* ≤ 100 V (upper axis). The basic processing parameter is varied over seven decades, 10^−2^ ≤ *P*_0_ ≤ 10^5^.

Of course, *P*_0_ typically is not similar with T-NIL and UV-NIL. As already addressed, the main parameter affecting its size is the viscosity *η_p_* of the imprinted material, which may differ by orders of magnitude. The interaction time *t_p_* may range from 0.5–30 min, depending on the system and the process. The surface tension has the smallest impact. Thus, there is an urgent need to know, at least approximately, the viscosity and the time during which instabilities may develop.

Considering viscosities typical of thermal imprint, *P*_0_ may range from about 0.2 to 50 m^3^/N^2^. This regime is marked in Figure 9 and assigned as ‘T-NIL’. It was considered that thermal instabilities dominate over van der Waals forces only with values of *P_th_* beyond ≈ 10^2^ K^2^m^3^/N^2^ (see Figure 7). This is the case from about Δ*T* ≈ 1 °C on.

Considering viscosities typical of UV-NIL, *P*_0_ may be in the range 70 to 2 × 10^4^ m^3^/N^2^ for imprint under electrostatic forces, again marked in Figure 9 and assigned as ‘el-UV-NIL’, dominating from *P_el_* ≈ 10^6^ V^4^m^3^/N^2^ on. This is the case with voltages of *U* ≥ 4 V.

When thermal imprint is combined with electrostatic forces, with or without intention, the range of viscosities (and thus *P*_0_) is similar to T-NIL; this is the regime assigned as ‘el-T-NIL’. If electrostatic instabilities are to dominate over van der Waals instabilities, again a value of *P_el_* ≈ 10^6^ V^4^m^3^/N^2^ has to be exceeded. This happens with more than 10 V only. However, thermal instabilities may also occur and may dominate at low voltages. Thus, both regimes apply in this case, ‘el-T-NIL’ and ‘T-NIL’ as well, with electrostatic and thermal instabilities, where the lower limit is the decisive one.

Figure 9 nicely shows that with the different imprint techniques the typical regimes are clearly separated, in terms of their risk of instabilities when varying the external control parameters, Δ*T* and *U*. The regions marked in grey indicate the conditions where the undulations are high enough to bridge the gap between mean polymer height and stamp ceiling within typical interaction times. Obviously, T-NIL is most critical, instabilities may develop over the whole range of temperature differences, 1 °C < Δ*T* < 100 °C. This is different with electrostatic fields. Defects have to be expected from 4V on in an electrostatic UV-NIL system; however, the voltage range allowed without inducing instabilities with a thermal process is substantially higher than with UV-NIL, due to the large difference in the viscosities.

Please note that instabilities due to van der Waals forces are not indicated in Figure 9. They would be present below the respective lower limits of the process-specific parameters, *P_th_* < 10^2^ K^2^m^3^/N^2^ and *P_el_* < 10^6^ V^4^m^3^/N^2^. Similarly, UV-NIL without an electric field cannot be indicated as it becomes limited by van der Waals instabilities only; there is no external control parameter available.

## 7. Summary

Based on experimental evidence, a guiding chart was developed to facilitate the choice of the initial layer thickness when imprinting periodic structures with a negligible residual layer. The strategy followed is ‘partial cavity filling‘, where a thin layer is printed to its full height so that isolated structures are obtained. When these isolated structures shall serve as a mask for subsequent etching with nanoimprint the lithography technique used, they have to be defect-free. To ensure this, geometric as well as thermodynamic limitations have to be overcome; the latter result from instabilities induced by van der Waals interactions, temperature gradients, or electrostatic forces. Practical use of the concept is encouraged by generalizing the underlying complex physical relationships and by presenting them in simplified form by means of graphs. These graphs can be used when a single, process-specific parameter is at hand. The construction of a tailor-made guiding chart applying to specific imprint situations was demonstrated and the processing window was discussed with T-NIL and UV-NIL, with and without electrostatic forces. Furthermore, measures to enlarge the defect-free processing window were addressed, emphasizing the stamp used and in particular its anti-sticking properties. Examples for an adequate choice of the initial layer thickness based on the respective processing window were given. To widen the applicability, the concept developed in detail with a linear, one-dimensional grating is adapted to a stamp featuring two-dimensional periodic structures.

## Figures and Tables

**Table 1:** Characteristic data of the imprint polymer used with our T-NIL experiments, PS, at typical processing temperatures (approximate values). *η_p_* = zero shear viscosity, *γ_p_* = surface tension, *θ* = contact angle to the stamp. Values for the viscosity and surface tension are taken from Refs. [58,60] and [61], respectively. The contact angle is determined from Young’s equation, in good agreement with experiments [45]. The surface energy of the stamp used to determine *θ* amounts to 10/15/20 mJ/m^2^, referring to excellent/good/limited anti-sticking properties, respectively.

	**25 °C**	**170 °C**	**190 °C**	**200 °C**	**250 °C**
*η_p_*/Pas	–	10^5^	2 × 10^4^	10^4^	10^3^
*γ_p_*/mJ/m^2^	41	33	31.5	30.5	27.5
*θ/*°	–	84/70/56	83/67/54	82/66/52	78/62/45

**Figure A1:**
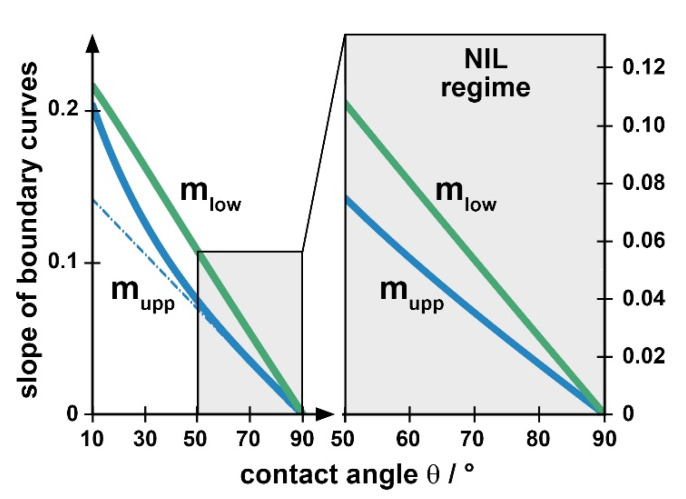
Non-dimensional slopes of the upper (blue) and lower (green) boundary for the formation of a horizontal meniscus in a circular or square/rectangular cavity as a function of the contact angle *θ*. With high contact angles the linear approximations are adequate (dash-dotted).

**Figure A2:**
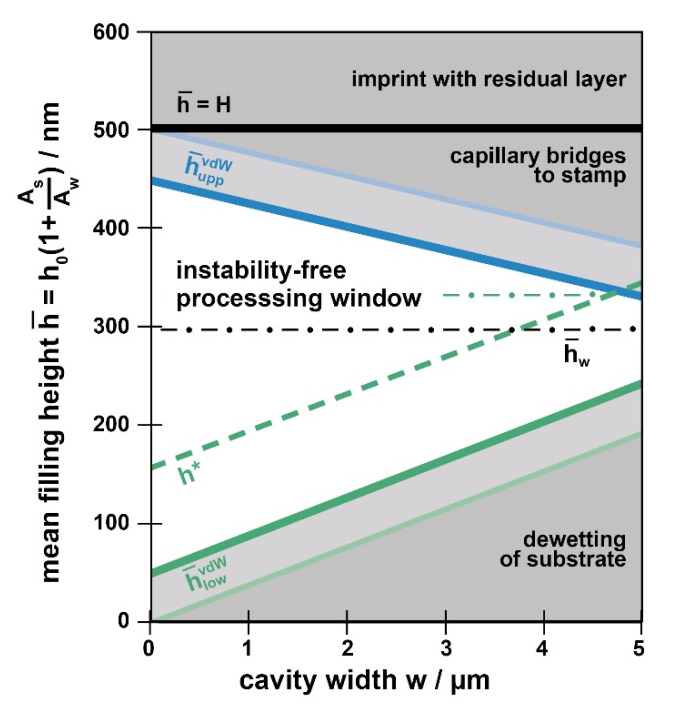
Example of a modified guiding chart for defect-free imprint with negligible residual layer similar to Figure 6, applying to a one-dimensional grating with isolated cavities. The processing window (bright) is limited by van der Waals instabilities, only.

## Appendix A. Periodicity

The periodicity incurring with instabilities differs with their origin [45,46,71,74]. Under conditions typical of nanoimprint, the following holds. Van der Waals instabilities exhibit the widest range of periods possible; periods of a few nanometers are found with narrow gaps but may extend into the millimeter range with wide gaps. With the limit taken above of $\bar{h}{\;}_{upp}^{vdW}$ = (*H* − 50 nm) the period is around 10 µm. With thermal and electrostatic instabilities, the range is smaller, 1–100 µm. When these instabilities occur together, thermal/electrostatic instabilities dominate with wide gaps (featuring long periods) and van der Waals instabilities dominate with narrow gaps (featuring small periods).

With our cavities we adopt the characteristics of a cavity resonator. When the period of a harmonic oscillation is larger than the dimension of the cavity, this oscillation will not show up (e.g., the case with the examples shown in Figure 2(a3–5), where the oscillation occurs in longitudinal direction but not in transversal direction, other than with Figure 2(b3,c1)). In the course of our simplified handling, we allocate thermal/electrostatic instabilities with a period of >10 µm and van der Waals instabilities with a period <10 µm, down to some 10 nm. Accordingly, van der Waals instabilities ‘see’ the cavity width and follow the width dependent geometric boundaries, ${\bar{h}}_{low}\left(w\right)$ and ${\bar{h}}_{upp}\left(w\right)$, but thermal/electrostatic instabilities do not; they are indicated in the guiding chart as a horizontal line, independent from the cavity width. As a consequence, the guiding chart introduced (Figure 6) covers cavities of some micrometer in width as an upper limit. With wider cavities the thermal/electrostatic limit would have to follow the cavity size dependence, similar to the van der Waals limit.

Of course, all instabilities will show up along the linear cavities, as addressed in connection with the reconfiguration of the polymer. With a length of linear cavities below ≈10 µm, thermal/electrostatic instabilities do not occur (see Appendix B).

## Appendix B. 2-Dimensional Gratings

The concept of the guiding chart developed for a linear, one-dimensional grating can be expanded to be used with stamps featuring a two-dimensional grating. The grating then consists of circular or square/rectangular geometries, arranged e.g., in a quadratic or hexagonal grid. Here the duty cycle of the stamp characterizing the material transport into the cavities is given by the respective area ratio *A_s_/A_w_* (rather than the ratio of the lateral geometries *s/w* with one-dimensional gratings). This area ratio reflects the size and distance of the periodic structures, as well as their lateral arrangement, the grid. Again, the guiding chart will be represented as the mean filling height, here according to $\bar{h}=h_{0}(1+A_{s}/A_{w}$), over the characteristic size of the cavities, *w_c_*, within a range of interest.

Furthermore, we have to distinguish between ‘grid-type’ stamps and ‘channel-type’ stamps. With a ‘channel-type’ stamp the cavities represent a connected network of channels and the polymer inside these channels behaves similar to a linear grating (compare Figure 6). Therefore, the characteristic width *w_c_* is the minimal distance between the elevated stamp structures. Accordingly, the geometric limitations are given by ${\bar{h}}_{upp}$ and ${\bar{h}}_{low}$, where Figure 5 and Equation (6) apply. All other boundaries are as before. Thus, the guiding chart for a ‘channel-type’ stamp resembles Figure 6; however, the ordinate is now $\bar{h}=h_{0}(1+A_{s}/A_{w}$), so that the initial layer height h_0_ corresponding to a certain filling level $\bar{h}$ reflects the two-dimensional grating, only. Often the situation is characterized by *A_s_/A_w_* << 1 so that the initial layer height *h*_0_ is not substantially smaller than the mean filling height $\bar{h}$.

With a ‘grid-type’ stamp the situation is different as isolated circular or square cavities exist. The characteristic width then is the diameter or the diagonal of the cavities (as already indicated by the experimental result of Figure 2(c4)). In that case, the upper and lower boundaries are again inclined lines in the guiding chart. However, their slope differs from Figure 5 as now the surface of the polymer forming a meniscus is a spherical segment (instead of the circular segment with the 1-dimensional grating). Calculating the respective geometric boundaries proceeds similar to Equations (3)–(5); the respective slopes of the upper and lower boundary are given in Figure A1, again as a function of the contact angle *θ*. Like before, a linear approximation holds in the range of the contact angles of interest,

(A1) $$m_{upp}\approx 0.16-1.8\cdot 10^{-3}\frac{\theta }{\deg}\approx \;\frac{2}{3}\;m_{low}.$$

As obvious from Equation (A1) and Figure A1, now the upper slope is the smaller one. Accordingly, a geometric guiding chart considering the slopes of Figure A1 (or Equation (A1)) applies.

To exemplify this situation, a guiding chart (similar to Figure 6) is given in Figure A2, considering a negative stamp with a two-dimensional grating, rectangular cavities (1 µm × 2 µm) at a distance of 1 µm, arranged in a square grid. Then the duty cycle amounts to *A_s_/A_w_* = 4/2 = 2 and the characteristic cavity width is *w_c_* ≈ 2.2 µm. Due to the limitation of the cavity in two dimensions, only van der Waals instabilities occur (see Appendix A).

With our example of Figure A2, a mean filling height of $\bar{h}\approx 300\;\mathrm{nm}$ could be adequate to avoid defects. Due to the duty cycle already high of *A_s_/A_w_* = 2 this requires an initial layer as low as 100 nm. This example clearly shows that despite the wide-looking processing window the realization of a defect-free imprint with isolated cavities may be hampered by preparing a very thin residual layer of high uniformity. Of course, this is due to the positive nature of the stamp. Generally, a similar issue exists with linear cavities, however typical duty cycles are not as high as with two-dimensional gratings.
